# Time Perspective Profile and Study Engagement

**DOI:** 10.3390/ejihpe15100191

**Published:** 2025-09-23

**Authors:** Zara-Anna Mathieu, Emilie Dujardin, Nicolas Noiret, Rébecca Shankland, Marie-Amélie Martinie

**Affiliations:** 1 CeRCA (Centre de Recherches sur la Cognition et l’Apprentissage), Université de Poitiers, Université de Tours, CNRS (Centre National de la Recherche Scientifique), 86000 Poitiers, France; zara.anna.mathieu@univ-poitiers.fr (Z.-A.M.); emilie.dujardin@univ-poitiers.fr (E.D.); nicolas.noiret@univ-poitiers.fr (N.N.); 2 DIPHE (Développement Individu Processus Handicap Education), Université Lyon 2, 69676 Lyon, France; rebecca.shankland@univ-lyon2.fr

**Keywords:** temporal perspective, balanced time perspective, study engagement

## Abstract

Academic dropout in French universities is significant. The lack of study engagement partly explains this phenomenon. Pursuing academic studies requires switching effectively among temporal orientations (past, present, and future). Although the relationships between study engagement and each temporal orientation have been studied, to the best of our knowledge, the association of all temporal profiles (present in all individuals) has not been considered in the relationship with study engagement. This study aimed to address this gap in the literature. In total, 451 French first- and second-year students enrolled in the humanities and social sciences Bachelor’s program completed a questionnaire including scales measuring time perspectives and study engagement. Using latent profile analyse, we obtained five profiles. We considered three of these as problematic profiles, including 40% of the students, and two had no problematic profiles. Among the latter, there is one in which 26% of the students are relatively oriented toward all temporal dimensions, and one balanced profile including 33% of the students. As expected, the balanced time perspective profile presented the highest study engagement scores, unlike past negative profiles, which showed lower scores. We discuss the implications of this new result for student academic success.

## 1. Introduction

The proportion of students who leave French universities without completing their first year is estimated at 31.8% (Ministère de l’Enseignement Supérieur et de la Recherche, 2020). Academic dropout is a complex and multifactorial phenomenon (e.g., parents’ social background, level of study, or working alongside studies; (Zaffran & Aigle, 2020) with one potential contributing factor being a lack of study engagement (Archambault et al., 2009). Enhancing study engagement, therefore, appears to be a relevant strategy to reduce academic dropout. Moreover, pursuing academic studies requires keeping in mind future goals, often for a very long term, while completing tasks in the present that may be unpleasant or boring in order to achieve the longer-term goals.

The relation between temporal orientations (i.e., past, present, and future) and study engagement has been mainly investigated using Zimbardo’s Time Perspective Inventory (ZTPI) (Zimbardo & Boyd, 1999). The ZTPI is designed to capture individual differences in time perception. This questionnaire distinguishes five temporal orientations: past positive (i.e., a positive view of the past, a warm and sentimental view of the past); past negative (i.e., a negative and aversive view of the past); present hedonistic (i.e., a focus on immediate pleasure with little consideration of the future); present fatalistic (i.e., an attitude of helplessness and a feeling of limited control over one’s life), and future (i.e., striving for future rewards and goals). Some temporal orientations are more desirable than others. For instance, present hedonist, present fatalist, and past negative are positively related to alcohol consumption, while a future orientation is negatively linked with alcohol consumption (Daugherty & Brase, 2010; Henson et al., 2006; Linden et al., 2014). A positive association has been observed between future time orientation and study engagement (Denovan et al., 2020; Hong, 2025; Liu et al., 2023; Peng & Zhang, 2022), suggesting that future time orientation could serve to detect or predict study engagement. Nevertheless, these studies did not consider the dynamic between past, present, and future, while time perspective is a continuous cognitive process that allocates attentional resources across different temporal perspectives in response to situational demands (Zimbardo & Boyd, 2008). To the best of our knowledge, the relationship between study engagement and the interplay of the past, present, and future has not yet been investigated. To this end, two objectives were set. First, we aimed to identify the temporal perspective of French students by considering the interplay between these perspectives using latent profile analyses. Secondly, we sought to examine the relationship between these profiles and study engagement, with the aim of identifying the profile that best predicts study engagement. Identifying the different latent profiles could be useful to detect the students who most need support during their studies.

### 1.1. Study Engagement and Time Perspective

Study engagement refers to a “positive, fulfilling, work-related state of mind that is characterized by vigour, dedication, and absorption in the activity” (Schaufeli et al., 2002, p. 72). High levels of energy and resilience during one’s studies refer to vigor, high inspiration and commitment refer to dedication, and being fully concentrated on and immersed in one’s study activities refers to absorption. Engagement encompasses three dimensions: emotional, behavioral, and cognitive. Positive emotional reactions to the academic study refers to the emotional dimension (Sinatra et al., 2015); the involvement in academic effort and persistence during the planning and attainment of learning activities refers to the behavioral dimension (Skinner et al., 2008), and the implication in learning and use of deep learning strategies refers to the cognitive dimension of study engagement (Xu et al., 2020). Study engagement is negatively linked to academic dropout and positively associated with academic success (Çali et al., 2024; Wang & Eccles, 2012), and well-being (Chaudhry et al., 2024; Denovan et al., 2020; Nolberto-Quispe et al., 2021). In sum, study engagement could be considered as a protective factor for students.

In academic settings, future time orientation appears to be particularly relevant for optimal academic outcomes, because it “provides the motivational resource needed to attain future goals” (Denovan et al., 2020, p. 1536). Indeed, being future-oriented is positively associated with self-regulated learning (de Bilde et al., 2011; Denovan et al., 2020), academic performance (Hong, 2025; Hong & Hanafi, 2024), school grades (Zimbardo & Boyd, 1999), and study engagement (Denovan et al., 2020; Haririzade et al., 2023; Hong, 2025; Liu et al., 2023; Peetsma, 2000; Peng & Zhang, 2022). These correlational studies are informative. Nevertheless, investigating only one specific temporal orientation or each of them independently is too restrictive. Indeed, in the same situation, individuals are not exclusively focused on one specific temporal orientation. In other terms, these correlational studies do not consider the interplay of the different time orientations, which are called time perspectives (TPs).

TP refers to the interplay between three temporal orientations present in individuals: past, present, and future. For Zimbardo and Boyd (1999, 2015), the psychological construction of the past and the anticipation of future events shape the concrete and practical representation of the present. TP is a pervasive filter that serves encoding, sorting, and recalling events to form expectations and objectives, and to construct scenarios. Therefore, it impacts behaviors and attitudes and may influence achievement, goal setting, and well-being. It is considered both as a disposition, when individuals have a habitual tendency to focus on one specific orientation, and as a state, when they focus on one orientation depending on situation features (Stolarski et al., 2018).

### 1.2. Balanced Time Perspective

Individuals can simultaneously manage all temporal orientations to varying degrees and switch from one to the other depending on personal resources and situational context. To achieve the most adaptive outcomes, Zimbardo and Boyd (1999) suggested that individuals should score high on past positive and future, while simultaneously scoring low on present hedonistic, present fatalist, and past negative. This profile would be optimal and corresponds to a balanced time perspective (BTP).

To operationalize BTP, four methods have been proposed. First, the cut-off scores method was used (Drake et al., 2008), which involves categorizing the five TP into low and moderate or high scores (i.e., below versus above the 33rd percentile). The second method consists of using cluster analyses that permit the identification of profiles. The third method consists of measuring the extent to which an individual’s ZTPI score profile deviates from an optimal score pattern associated with a BTP (Stolarski et al., 2011). It is widely used to operationalize BTP (for a review of research, Stolarski et al., 2020). The optimal score values are justified by Stolarski et al. (2011) as follows: “Following Zimbardo and Boyd’s proposal www.thetimeparadox.com/surveys (accessed on 25 March 2025) and based on Zimbardo and Boyd’s collective cross-cultural database, we defined a ‘high’ score on past positive as 4.60, a ‘moderately high’ score on present hedonism and future as 3.90 and 4.00 respectively, and ‘low’ score on past negative and present fatalism as 1.95 and 1.50 respectively” (p. 354). The smaller the DBTP value—reflecting minimal deviation from optimal profile—the closer an individual’s score profile is to BTP. Finally, a little-used method consists of establishing latent profiles. Latent profile analysis (LPA) is a categorical latent variable modeling approach that is used to identify a number of profiles in a sample and estimate the probability of membership of each profile by analyzing different configurations of several variables (see Spurk et al., 2020).

Some authors (Zimbardo & Boyd, 1999; Boniwell & Zimbardo, 2004) suggested that individuals with BTP should show the most positive outcomes. Regarding mental health, this hypothesis has been tested with different methods to operationalize BTP. It has been observed that with the cut-off scores, a BTP is crucial to have high levels of well-being; participants with moderate or high BTP scores reported more positive states than those with low BTP scores (Drake et al., 2008). With the DBTP value, the lesser individuals deviate from the BTP profile, the higher their level of well-being (Fuentes et al., 2022; for a review of research, see Stolarski et al., 2020), and greater resilience regarding mental health problems such as anxiety, stress, and depression (Griffin & Wildbur, 2020). Also, with latent profile analyses (LPA), the balanced latent profile was linked with less drinking behavior (Braitman & Henson, 2015). Recently, Kossewska et al. (2023) observed that individuals who scored high on future, present hedonistic, past positive, and low on present fatalistic and past negative reported the least educational, social, and mental health problems. This latent profile is not completely balanced. Nevertheless, it was obtained in a specific context. In fact, the study was carried out during the COVID-19 pandemic. In this context, in which death was salient, it is not surprising to observe that students were more oriented towards a hedonistic present. Taken together, these results support the view of Zimbardo and Boyd (1999). Nevertheless, with cluster analyses, some results do not support this view. For instance, it has been reported that adolescents with a future profile displayed the most positive outcomes (i.e., problematic drinking behaviors) (McKay et al., 2014), while students with a balanced profile displayed the most positive outcomes (i.e., well-being). These differences in results can be explained by differences in populations (adolescents or students) and differences in outcomes (problematic drinking behaviors or well-being) (Boniwell et al., 2010). Moreover, the cut-off-scores method, cluster analyses, and DBPT formula have been compared. It has been observed that DBTP was the best predictor of subjective well-being (Zhang et al., 2013).

Although in the field of mental health, the DBPT formula is the most widely used, it is methodologically highly questionable. Indeed, the values of optimal scores have been established on the collective cross-cultural database without demographic information provided, while the age of participants is a crucial element to consider. In fact, TP is a psychological process that develops throughout life (Steinberg et al., 2009) and, for instance, for teenagers, it is influenced by interactions with significant others such as parents or teachers (Luyckx et al., 2010). Jankowski et al. (2020) point out that the values of optimal scores are identical for each individual and are established arbitrarily. In fact, the values correspond to the percentile distribution (10th, 80th, or 90th) from the large cross-cultural database of Zimbardo and Boyd (2008). Moreover, in some studies, TP subscales were more linked with psychological outcomes than DBTP (see, for instance, Stolarski, 2016). This calls into question the usefulness of DBTP. Furthermore, the cut-off scores are methodologically highly questionable. More precisely, the cut-off criteria were selected arbitrarily, and nothing permits us to conclude that they are optimal (Boniwell et al., 2010). Moreover, concerning cluster analyses, one of its limits is that studies provide differences in temporal profiles depending on the country. For instance, a profile similar to the BTP has been observed among an Australian sample and not among British, American, and Slovenian samples (McKay et al., 2018). Moreover, Sircova et al. (2015) observed a balanced profile in 24 countries (17 from Europe, 3 from Asia and America, and 1 from Africa), with the prevalence of a balanced profile for some countries, such as Estonia and Israel, and a prevalence of a present-orientation profile for a French sample.

In light of the various criticisms of the DBTP formula and the cut-off scores method, the latent profile analyses seem to be the most relevant (see Spurk et al., 2020). Moreover, comparatively to cluster analysis, latent profile analyses offer several methodological advantages. For instance, latent profiles assign individuals to profiles based on probabilities, while cluster analyses assign each individual to a single cluster, without uncertainty. So, latent profiles allow for soft classification, which better reflects real-world ambiguity in group membership. Moreover, latent profiles are based on underlying statistical models (e.g., mixed models), enabling inference, hypothesis testing, and model fit assessment, while clusters rely on distance-based algorithms with no statistical model behind them. With latent profile analyses, statistical indices like AIC/BIC can be used to choose the number of latent profiles that can include measurement error in the modeling process, while clusters treat the data as exact and noise-free.

### 1.3. Purpose of This Research

In the present research, we investigated the relation between the dynamic process of temporal perception and study engagement among French students. To the best of our knowledge, this relationship has not yet been investigated in any population. Given that study engagement promotes positive outcomes such as academic achievement (Lei et al., 2018; Serrano & Andreu, 2016) and well-being (Chaudhry et al., 2024; Denovan et al., 2020; Nolberto-Quispe et al., 2021), and that the BTP should lead to the most positive outcomes (Zimbardo & Boyd, 2008), we hypothesized that the balanced latent profile would be best associated with study engagement. To test this hypothesis, we 1/identified among French students the different TP latent profiles, 2/investigated the relation between TP latent profiles and study engagement.

## 2. Method

### 2.1. Ethics Approval

This research received no funding. It was conducted in accordance with the principles of the Declaration of Helsinki and was approved by the local university’s Ethics Committee (CER-TP 2024-02-05). Written consent was obtained from all participants. The data are publicly available on the Open Science Framework and can be accessed at https://osf.io/nxvty/?view_only=1e68ed2df202439c84d75e9eee5a3acb (accessed on 8 September 2025).

### 2.2. Participants

The study was conducted in a French university among first- and second-year students in the humanities and social sciences Bachelor’s program (psychology). The sample total was composed of 451 participants (412 females). The participants were all native French speakers and aged 18 or older (Mage = 19.2, SEage = 2.71).

### 2.3. Procedure

The participants took part in this study in exchange for course credits. The study was performed in a lab room in collective sessions with 8 participants at a time. Each participant was seated in front of a computer, 2 m apart, and completed a computerized questionnaire elaborately programmed on OpenSemame 0.24 (Mathôt et al., 2012). In this questionnaire, the study engagement and time perspective scales were counterbalanced across participants.

### 2.4. Measures

#### 2.4.1. Study Engagement

The Study Engagement Scale (Salmela-Aro & Upadaya, 2012) is composed of 9 items. Three items assess absorption (e.g., “Time flies when I’m studying”), three items vigor (e.g., “When I’m studying, I feel mentally strong”), and three items dedication (e.g., “My studies inspire me”). All items were rated on a 5-point Likert-type scale ranging from 1 (not at all true for me) to 5 (totally true for me). The plausibility of the overall model was assessed based on several goodness-of-fit measures, such as Comparative Fit Index (*CFI*), Tucker–Lewis Index (*TLI*), the Root-Mean Square Error of Approximation (RMSEA), and Standardized Root Mean Squared (*SRMS*) with a 90% confidence interval. Guidelines for values indicative of good fit are *CFI* and *TLI* equal or greater than 0.90, and RMSEA and SRMR less than or equal to 0.08 (Hu & Bentler, 1999). With the 3 dimensions of study engagement (i.e., absorption, vigor, and dedication), the goodness of fit indices indicated that the model had no satisfactory fit: *CFI* = 0.95, *TLI* = 0.92, *RMSEA* = 0.10, *SRMR* = 0.05. Modification indices analysis suggested removing the items “When I get up in the morning, I feel like going to class” and “I get carried away when I am studying”. In this case, the model had a satisfactory fit, *CFI* = 0.99, *TLI* = 0.97, *RMSEA* = 0.07, and SRMS = 0.03. Internal consistency was satisfactory for absorption (*r* = 0.43), vigor (*r* = 0.62), and dedication (ω = 0.88). Given that the three dimensions of study engagement were highly intercorrelated (*p* < 0.001), mean score of study engagement was calculated.

#### 2.4.2. Time Perspective

We used the French short version of the Zimbardo Time Perspective Inventory (ZTPI) (Fritsch & Cuervo-Lombard, 2022) to assess time perspective. The scale is composed of 15 items, with 3 items assessing each time orientation: past positive (e.g., “thinking about my past gives me pleasure”), past negative (e.g., “I think about the bad things that happened to me in the past”), present hedonistic (e.g., “I always find myself driven by the excitement of the moment”), present fatalistic (e.g., “Since what must happen will happen, it really doesn’t matter what I do”), and future (e.g., “I bring my projects to fruition on time, taking things one step at a time”). All items were rated on a 5-point Likert-type scale ranging from 1 (not at all true for me) to 5 (totally true for me). The goodness of fit indices indicated that the model did not have a satisfactory fit: *CFI* = 0.91, *TLI* = 0.89, *RMSEA* = 0.06, *SRMR* = 0.06. Modification index analysis suggested removing the item “I no longer enjoy doing things if I have to think about objectives, consequences and results”. In this case, the model had a satisfactory fit, *CFI* = 0.95, *TLI* = 0.93, *RMSEA* = 0.05, and *SRMR* = 0.05. Internal consistency was satisfactory for past negative (ω = 0.76), past positive (ω = 0.75), present fatalistic (r = 0.44), present hedonistic (ω = 0.66), and future (ω = 0.70). The mean score for each temporal dimension was calculated.

## 3. Results

### 3.1. Analytic Plan

We used the RStudio 2024.12.1. (Posit Team, 2024) and Mplus 8.10 software (Muthén & Muthén, 1998–2017) to process the results. Correlation analyses were conducted to examine relations among variables using Pearson’s r. To investigate the existence of temporal perspective profiles, we conducted a latent profile analysis (LPA, package “MplusAutomation”, Hallquist & Wiley, 2018) to identify distinct profiles of temporal perspective with the Mixture Modeling Method, and we used robust maximum likelihood estimation (MLR) estimator because the data did not follow a normal distribution (Asparouhov & Muthén, 2005). Finally, to address our last research question, concerning the relationship between each profile and engagement, we employed the Bolck–Croon–Hagenaars (BCH) method to assess whether profile membership predicted academic engagement (Bakk & Vermunt, 2016). Specifically, we conducted equality tests to compare the mean engagement scores of participants across all latent profiles. Its main advantage lies in the third step, where an analysis of variance is performed using individual weights derived from classification uncertainty. These weights serve as an improved latent profile indicator, offering a more accurate alternative to relying solely on modal class assignment.

### 3.2. Descriptive Statistics

First, the data were checked to ensure no missing values or anomalies in the cases. Six participants with aberrant or missing data were excluded. Age data was missing for 15 participants; therefore, we imputed the mean value of 19.2 years. Means, standard deviations, and correlations are presented in Table 1. Correlation analyses showed that study engagement was positively correlated with past positive (*p <* 0.001), present hedonistic (*p <* 0.01), and future (*p <* 0.001), and negatively correlated with past negative (*p <* 0.001) and present fatalistic (*p <* 0.01) (see Table 1).

### 3.3. Latent Profile Analysis

The LPA was conducted to determine the number of profiles related to students’ temporal perspective, using factor scores previously extracted from a confirmatory factor analysis of the ZTPI scale (with five indicators: past positive, past negative, present fatalistic, present hedonistic, and future). Means were allowed to be different across classes, and residual variance was allowed to be equal but freely estimated. To select the optimal number of profiles, we have considered the theoretical meaning of the profiles and the need to obtain a statistically satisfactory solution (see Spurk et al., 2020). Different criteria were applied to evaluate the fit of the LPA model and to select the best reading profile models: 1/Information criteria: Akaike Information Criterion (*AIC*), Bayesian Information Criterion (*BIC*), and sample-size adjusted BIC (*SSABIC*). The model with the lowest values on these criteria was selected as the best-fitting model. 2/Entropy: Values approaching 1 indicate high classification accuracy. 3/Statistical tests: Parametric bootstrap likelihood ratio test (*BLRT*), Vuong–Lo–Mendell–Rubin test (VLMR), and adjusted Lo–Mendell–Rubin test (A-LMR). A *p*-value inferior to 0.05 suggests that adding an extra profile improves model fit. 4/Proportion of participants in each profile: profiles with at least 5% of the sample were considered meaningful.

Table 2 provides the fit statistics for possible latent profile structures. Although the likelihood-based indices (*AIC*, *BIC*, *CAIC*, and *SSABIC*) continue to decrease as the number of classes increases, the rate of improvement slows after five classes. The entropy value at five classes (0.78) indicates good classification quality. Moreover, the VLMR-LRT and ALMR tests are non-significant beyond four classes, suggesting that adding more profiles does not yield statistically significant improvement. Considering the balance between model fit, classification accuracy, and theoretical interpretability, the five-class solution was selected as the optimal model.

Table 3 displays the results of the profile analysis. Based on the above criteria, we stopped at a 5-profile structure. Although VLMR—LRT and ALMR are not significant at profile 3, indicating that we can stop adding profiles, the indices are significant at a 4-profile structure, but not at larger numbers of profiles. Other arguments led us to choose the 5-profile solution as it has a lower AIC, BIC, and SSABIC than the 4-profile solution, and has a higher entropy index. Although one profile has less than 5% of members (4.6%, Profile 1), we consider this profile as pertinent theoretically, as discussed below.

**Figure 1:**
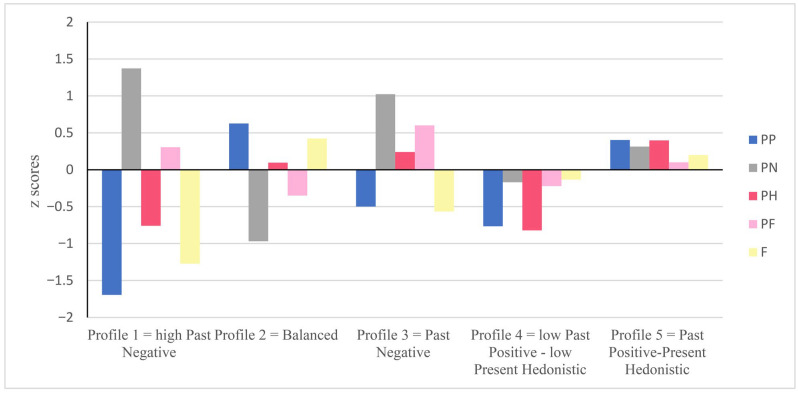
Mean scores of the five ZTPI dimensions (PP: past positive; PN: past negative; PH: present hedonistic; PF: present fatalistic; F: future) for the five temporal perspective profiles.

Profile 1—High Past Negative: It is characterized by a past negative score well above the mean and a past positive score at the opposite extreme, well below the mean. It includes 21 students (4.60%). We kept this profile because it is not theoretically abstract. Indeed, the undesirable orientations (past negative and present fatalistic) oppose the desirable orientations (past positive, present hedonistic, and future). Profile 2—Balanced: It is characterized by high scores on past positive and future, a present hedonistic score slightly above the mean, and lower-than-average scores on past negative and present fatalistic. It includes 149 students (33%). This profile corresponds to the “balanced” temporal perspective described in the scientific literature by Zimbardo and Boyd (1999). Profile 3—Past Negative: It is characterized by a very high past negative score relative to the other dimensions, along with below-average scores on past positive and future. It includes 82 students (18%). Profile 4—Low Past Positive–Low Present Hedonistic: All temporal perspective dimensions are below the mean, with particularly low scores for past positive and present hedonistic. It includes 79 students (17.50%). Profile 5—Past Positive—Present Hedonistic: It reflects students who are relatively oriented toward all temporal dimensions. Past positive and present hedonistic are slightly above the mean, past negative and future are lower, and present fatalistic is slightly above the mean. It includes 120 students (26.60%). Figure 1 illustrates the selected 5-profile solution, and Table 4 shows the estimated parameters for the 5-profile model.

### 3.4. Bolck–Croon–Hagenaars—Differences in Sex, Age, and Engagement Across the Five Temporal Perspective Profiles

Differences in sex, age, and academic engagement between the five temporal perspective profiles were examined using the BCH method, which allows for accurate comparison of distal continuous and categorical outcomes while accounting for classification uncertainty in latent profile analysis (see Table 5). This approach ensured robust group comparisons across profiles without influencing the latent class formation.

#### 3.4.1. Sex Differences Across Profiles

Sex distribution significantly differed across the five temporal perspective profiles, as indicated by the BCH omnibus test (χ^2^ (4) = 41.94, *p* < 0.001). Pairwise comparisons showed that the high past negative profile consisted of 100% women, which was significantly different from the balanced (89.8%, *p* < 0.001), past negative (91%, *p* = 0.018), low past positive–low present hedonistic (92.7%, *p* = 0.023), and past positive–present hedonistic profiles (90.8%, *p* = 0.001). No other significant sex differences were observed between the profiles.

#### 3.4.2. Age Differences Across Profiles

Age differed significantly between the temporal perspective profiles, according to the BCH omnibus test (*χ*^2^ (4) = 17.25, *p* = 0.002). Pairwise comparisons revealed that the high past negative profile (*M*_age_ = 18.46) was significantly younger than the balanced profile (*M*_age_ = 19.46, *p* = 0.016). There was a trend for the low past positive–low present hedonistic profile (*M*_age_ = 19.63) to be older than the high past negative profile (*p* = 0.057). The balanced profile was also significantly older than the past negative profile (*M*_age_ = 18.52, *p* = 0.003), and the past negative profile differed marginally from the low past positive-low present hedonistic profile (*p* = 0.050). Additionally, the past negative profile was significantly younger than the past positive–present hedonistic (*M*_age_ = 19.10 years). No other age differences were statistically significant.

#### 3.4.3. Engagement Differences Across Profiles

Academic engagement significantly differed across the temporal perspective profiles, as indicated by the BCH omnibus test (*χ*^2^ (4) = 89.32, *p* < 0.001). Pairwise comparisons based on the BCH-adjusted means revealed that the high past negative profile reported significantly lower engagement levels than the balanced profile (*p* < 0.001), 4 (*p* = 0.014), and 5 (*p* < 0.001). The balanced profile, which showed the highest engagement, differed significantly from all the other profiles: the high past negative profile (*p* < 0.001), past negative profile (*p* < 0.001), low past positive–low present hedonistic profile (*p* < 0.001), and past positive–present hedonistic profile (*p* < 0.001). Moreover, Profile 3 had significantly lower engagement than Profiles 4 (*p* < 0.01) and 5 (*p* < 0.001). No other pairwise comparisons reached statistical significance. These results suggest that temporal perspective profiles are meaningfully associated with students’ levels of academic engagement.

## 4. General Discussion

The main aim of the present study was to identify 1/latent profiles of TP among French students and 2/the latent profile that is the best associated with study engagement. Based on Zimbardo and Boyd’s (2008) view, we expected that the profile best associated with study engagement is balanced. Our results highlight the existence of five profiles, including a balanced profile. As expected, our results showed that the balanced profile is the best associated with study engagement scores. To the best of our knowledge, it is the first time that a study has shown the relationships between study engagement and specific temporal orientation profiles.

About the profiles, we observed that proportion of students in the profiles is variable. More precisely, the balanced profile encompasses 33%; past positive—present hedonist 26.60%; past negative 18%; low past positive–low present hedonist with 17.50% and high past negative 4.60% of the students. In sum, our results show that around 60% of the students have no problematic profile (i.e., balanced and past positive—present hedonist). On the contrary, 22.6% of the students have TP oriented toward high past negative and high present fatalistic, and 17.5% have TP oriented toward low past positive and low present hedonistic. This raises questions about the well-being of these students.

Two types of analyses have been used to identify the BTP profile in the literature: cluster analysis and latent profile analysis. Nevertheless, the results obtained with these two analyses are inconsistent. More precisely, with cluster analysis, McKay et al. (2014) observed that adolescents (ages 12–16) with a BTP profile did not report the most adaptive outcomes. More precisely, for adolescents with a BTP and a past negative profile, the probability of being a problematic or a moderate drinker is identical, while it is smaller for those with a future profile. McKay et al. (2014) suggested that among adolescents, the future profile would be more optimal, while the BTP profile would be more optimal among young adults and adults. Nevertheless, McKay et al. (2018) constated that Australian students with a BTP profile did not differ from those with a past negative–fatalist profile on problematic alcohol use, depression, anxiety, optimism, and self-esteem. They reported lower self-esteem and optimism scores and higher depression than those with an ambivalent profile. In sum, contrary to Boniwell and Zimbardo’s (2004) view, a BTP profile identified by cluster analysis appears not to lead to the most adaptive outcomes, while a BTP based on latent profile analysis is linked with less engagement in drinking behavior (Braitman & Henson, 2015) and with higher study engagement in our study. Moreover, with a latent profile not completely balanced—but obtained in a specific context (i.e., participants tested before or during the COVID-19 pandemic)—fewer educational, social, and mental health problems were observed (Kossewska et al., 2023). A balanced profile based on latent profile analysis appears to be more theoretically adequate for Zimbardo and Boyd’s (1999) hypothesis than a balanced profile based on cluster analysis. On a large sample, a latent profile analysis appears to be more appropriate than a cluster analysis. Indeed, contrary to a cluster analysis based on a specific sample, with a latent profile analysis, the probability of belonging to each profile is calculated for each participant. The participant is assigned to the profile to which he or she is most likely to belong (Spurk et al., 2020).

### 4.1. Implications

Identifying the different latent profiles is useful to detect the students who most need support (i.e., students at-risk) in order to increase their study engagement—a crucial factor for academic success (Çali et al., 2024; Wang & Eccles, 2012), and well-being (Chaudhry et al., 2024; Denovan et al., 2020; Nolberto-Quispe et al., 2021). Once at-risk students are identified, it is possible to support them in different ways. For unbalanced profiles, if temporal orientations do not reveal a trait, it can be suggested to focus students on Future orientation because 1/it is an important element of BTP, and 2/it is positively associated with Past Positive (e.g., Denovan et al., 2020; Zhang et al., 2013), another important dimension of BTP.

To increase future orientation, it is possible to prime them on a future-oriented mindset; they prioritize more distal goals than proximal goals (Dreves & Blackhart, 2019). Also, it is possible to improve self-efficacy. It is a malleable skill defined as the *“beliefs in one’s capabilities to organize and execute courses of action required to produce given attainments”* (Bandura, 1997, p. 3). In academic contexts, self-efficacy is positively associated with future orientation (for a review see Kooij et al., 2018), and negatively with past negative (Zebardast et al., 2011), and present fatalistic (Taylor & Wilson, 2019; Zebardast et al., 2011). How individuals interpret their past experiences (mastery experiences), vicarious experiences, somatic/affective states (i.e., stress, fatigue, anxiety, and mood), and social persuasion (positive feedback from family, peers, and teachers) are major sources of self-efficacy (Bandura, 2001), and the most powerful is mastery experience (see meta-analysis by Byars-Winston et al., 2017). Positive feedbacks allow students to have information about their mastery of the task (Schunk & Swartz, 1993; Zumbrunn et al., 2016). Moreover, focusing on the strengths and factors that contributed to past achievement and students’ strengths increases mastery experience (Ajzen, 2002).

Finally, BTP is considered as a specific expression of the ability to manage internal states in a constructive way (Kashdan & Rottenberg, 2010). This involves cultivating psychological flexibility, which refers to the capacity to act consistently with one’s values while remaining connected to the present moment, even in the face of difficult feelings, sensations, and thoughts (Hayes et al., 2006). Increasing psychological flexibility is relevant in educational settings, as it is 1/positively associated with academic self-efficacy (Martinie & Shankland, 2024; Schéle et al., 2021)—a crucial predictor for student achievement (see meta-analysis of Multon et al., 1991), 2/positively predicts study engagement (Martinie & Shankland, 2024), 3/is positively associated with past positive and negatively with past negative and present fatalistic orientations (Pyszkowska & Rönnlund, 2021). Psychological flexibility is a malleable skill that can be developed through specific interventions based on acceptance and commitment therapy (ACT) principles (Hayes et al., 2006). Universities could be encouraged to lead interventions based on ACT principles among their students, as they have been shown to improve study engagement and psychological capital (Fang & Ding, 2020; Grégoire et al., 2018, 2019).

### 4.2. Limitations

The present study has several limitations. Our sample consisted exclusively of French students enrolled in humanities and social sciences (HSS). As such, our findings should be interpreted as specific to this population of students, which has a higher tendency to procrastinate (Martinie et al., 2022 for a study with French students enrolled in HSS; Nordby et al., 2017), which negatively impacts their study engagement (Afrashteh & Janjani, 2024). In future work, it could be interesting to compare the time perspective of these populations of students to that of other populations, such as STEM students, in order to better understand the role of temporal profiles and causes of dropout. In the same line, these results could be completed by the study of other psychological characteristics such as mood, anxiety, personality traits, or metacognitive processes, which could be particularly associated with specific profiles (Stolarski et al., 2020). We observed an effect of age on profiles that suggests that profiles could be evolving over time. Therefore, it will be necessary to consider time in establishment of the profile. Indeed, it could be relevant to test the profile structure from the same sample at several time points and examine the extent to which individuals could transition between different profiles. Furthermore, we observed differences according to sex. Nevertheless, it is worth mentioning that our population was an almost entirely female population. To generalize our results, future research should investigate other student populations and consider age and sex. Moreover, our study did not consider family socioeconomic status, while it is positively associated with study engagement (Fullarton, 2002; Tomaszewski et al., 2020). Future research should consider this factor to establish latent profiles of TP and other sociodemographic information, such as family status or employment. Finally, the association between latent profile and study engagement does not allow for any causal conclusions about the observed relationships.

### 4.3. Conclusions

Our results highlight five latent profiles among French students. Two are really problematic, in which students are highly focused on past negative experiences and are little oriented toward the future. Furthermore, we observed another problematic profile in which students show a low level on all orientations, and particularly on past positive and present hedonist. These three problematic profiles suggest that 40% of the students could be at-risk. Moreover, we observed two non-problematic profiles. One in which students are relatively oriented toward all temporal dimensions, including 26.60% of the students, and one balanced profile, including 33% of the students. As expected, the balanced profile was best associated with study engagement, a central factor in academic performance (Lei et al., 2018; Serrano & Andreu, 2016), and student well-being (Chaudhry et al., 2024; Denovan et al., 2020; Nolberto-Quispe et al., 2021). Therefore, identifying students’ profiles could be a means of detecting at-risk students in order to support them more specifically during their studies.

## Figures and Tables

**Table 1:** Means, standard deviations, internal consistencies, and intercorrelations between ZTPI dimensions and academic engagement.

	*M*	*SD*	1	2	3	4	5	6	7	8	9
1. Past Positive	9.58	2.95	(*ω* = 0.75)								
2. Past Negative	9.18	3.36	−0.289 ***	(*ω* = 0.76)							
3. Present Hedonist	9.31	2.63	0.236 ***	0.041	(*ω* = 0.66)						
4. Present Fatalistic	6.64	2.55	−0.110 *	0.299 ***	0.059	(*r* = 0.44)					
5. Future	9.75	2.73	0.236 ***	−0.245 ***	0.056	−0.157 ***	(*ω* = 0.70)				
6. Engagement	29.6	6.98	0.195 ***	−0.272 ***	0.139 **	−0.238 ***	0.524 ***	*(ω* = 0.87)			
7. Devotion	11.5	2.72	0.147 **	−0.225 ***	0.099 *	−0.211 ***	0.410 ***	0.884 ***	(*ω* = 0.88)		
8. Vigor	8.54	2.53	0.189 ***	−0.282 ***	0.166 ***	−0.189 ***	0.465 ***	0.867 ***	0.632 ***	(*r* = 0.62)	
9. Absorption	9.63	2.65	0.183 ***	−0.216 ***	0.107 *	−0.231 ***	0.514 ***	0.899 ***	0.698 ***	0.681 ***	(*r* = 0.43)

*Note*. *M* = mean; *SD* = standard deviation; *ω* = McDonald’s Omega; *r* = Pearson’s r (for bivariate correlation); * *p* < 0.05, ** *p* < 0.01, and *** *p* < 0.001.

**Table 2:** Goodness of fit statistics for model comparison (standardized scores).

Model	Log-Likelihood	Best Log Replicated	Parameters	Scaling	AIC	BIC	SSABIC	Entropy	VLMR-LRT (p)	ALMR (p)	BLRT (p)
1 Class	−2877.69	1	10	0.862	5775.38	5816.49	5784.76	-	-	-	-
2 Class	−2754.28	1	16	1.354	5540.57	5606.35	5555.57	0.67	0.030	0.032	0.00
3 Class	−2717.29	1	22	1.547	5478.59	5569.04	5499.22	0.72	0.403	0.409	0.00
4 Class	−2663.32	1	28	1.086	5382.65	5497.77	5408.91	0.77	0.000	0.000	0.00
5 Class	−2644.06	1	34	1.140	5356.11	5495.90	5388.00	0.78	0.214	0.221	0.00
6 Class	−2626.47	1	40	1.261	5332.95	5497.40	5370.46	0.77	0.489	0.497	0.00
7 Class	−2606.66	0	46	1.291	5305.33	5494.46	5348.47	0.80	0.367	0.373	0.00
8 Class	−2591.33	0	52	1.330	5286.66	5500.46	5335.43	0.81	0.524	0.529	0.00

*Note*. AIC, Akaike Information Criteria; BIC, Bayesian Information Criteria; SSABIC, Sample Size-Adjusted Bayesian Information Criteria; VLMR, Vuong–Lo–Mendell–Rubin likelihood ratio test; A-LMR, Lo–Mendell–Rubin adjusted likelihood ratio test; BLRT, parametric bootstrapped likelihood ratio test.

**Table 3:** Distribution of temporal perspective dimensions by latent profile and percentage of students.

	Profile 1	Profile 2	Profile 3	Profile 4	Profile 5
Past Positive	−1.694	0.625	−0.5	−0.766	0.403
Past Negative	1.372	−0.97	1.023	−0.17	0.314
Present Hedonist	−0.759	0.095	0.24	−0.822	0.398
Present Fatalistic	0.306	−0.35	0.599	−0.222	0.101
Future	−1.272	0.423	−0.566	−0.134	0.2
N	21	149	82	79	120
Pourcentage	4.60%	33%	18%	17.50%	26.60%

*Note*. Values represent standardized means (z-scores) of each ZTPI dimension across the five latent profiles. Profile 1 = high past negative profile; Profile 2 = balanced profile; Profile 3 = past negative profile; Profile 4 = low past positive—low present hedonistic profile; Profile 5 = past positive–present hedonistic profile.

**Table 4:** Estimated parameters for the 5-profile model.

	*b (s.e.)*	*S.E.*	*Est./SE*	*p*
Profile 1 (4.6%)				
SPP	−1.69	0.136	−12.491	<0.001
SPN	1.37	0.117	11.755	<0.001
SPH	−0.76	0.470	−1.614	0.107
SPF	0.31	0.317	0.965	0.334
SF	−1.27	0.256	−4.967	<0.001
Profile 2 (33%)				
SPP	0.63	0.060	10.328	<0.001
SPN	−0.97	0.048	−20.429	<0.001
SPH	0.10	0.073	1.300	0.194
SPF	−0.35	0.069	−5.106	<0.001
SF	0.42	0.065	6.556	<0.001
Profile 3 (18%)				
SPP	−0.50	0.226	−2.211	0.027
SPN	1.02	0.130	7.847	<0.001
SPH	0.24	0.112	2.148	0.032
SPF	0.60	0.185	3.244	0.001
SF	−0.57	0.130	−4.343	<0.001
Profile 4 (17.5%)				
SPP	−0.77	0.116	−6.612	<0.001
SPN	−0.17	0.100	−1.708	0.088
SPH	−0.82	0.114	−7.186	<0.001
SPF	−0.22	0.098	−2.261	0.024
SF	−0.13	0.112	−1.201	0.230
Profile 5 (26.6%)				
SPP	0.40	0.095	4.235	<0.001
SPN	0.31	0.089	3.534	<0.001
SPH	0.40	0.093	4.270	<0.001
SPF	0.10	0.108	0.929	0.353
SF	0.20	0.111	1.796	0.073

*Note*. N = 451; *b*: unstandardized coefficient; *S.E.*: standard error; Profile 1 = high past negative profile; Profile 2 = balanced profile; Profile 3 = past negative profile; Profile 4 = low past positive—low present hedonistic profile; Profile 5 = past positive–present hedonistic profile.

**Table 5:** Characteristics of the mixture regression profiles on covariates.

Variable	(*N* = 451)	M	SD	Profile 1	Profile 2	Profile 3	Profile 4	Profile 5	Global Test	Summary of Significance Tests
% Female	91.35%	–	–	100.00%	89.8%	91.0%	92.7%	90.8%	χ^2^ (4) = 41.94, *p* < 0.001	1 > 2 = 3 = 4 = 5
Age	–	19.2	2.71	18.46	19.46	18.52	19.63	19.10	χ^2^ (4) = 17.25, *p* = 0.002	1 = 4 = 5; 3 < 4 = 5 = 2; 2 > 1 = 3
Engagement	–	29.6	6.98	−0.73	0.46	−0.60	−0.14	0.08	χ^2^ (4) = 89.32, *p* < 0.001	1 = 3 < 5 = 4 > 2

*Note*. Raw scores were used for the descriptive statistics of the engagement and age variables. Percentages of females per profile are reported for each profile. Multiple comparisons are based on post hoc tests following the latent profile analysis with covariates. *M* = mean; *SD* = standard deviation; *p* = probability value. Profile 1 = high past negative profile; Profile 2 = balanced profile; Profile 3 = past negative profile; Profile 4 = low past positive—low present hedonistic profile; Profile 5 = past positive–present hedonistic profile.

## Data Availability

The data are publicly available on the Open Science Framework and can be accessed at https://osf.io/nxvty/?view_only=1e68ed2df202439c84d75e9eee5a3acb (accessed on 8 September 2025).
